# Supplementary material for: Peptide−Calcium Chelate from Antler (Cervus elaphus) Bone Enhances Calcium Absorption in Intestinal Caco-2 Cells and D-gal-Induced Aging Mouse Model
Source: Nutrients. 2022 Sep 10;14(18):3738. doi: 10.3390/nu14183738 (PMC9504974; doi:10.3390/nu14183738)
Supplement: Supplementary file 1 [file nutrients-14-03738-s001.zip › nutrients-1800841-supplementary.pdf]

Supplementary data

Table S1. Effects of D-gal administration on liver oxidative stress of male mice.

Study model	Age (weeks)	D-gal			Major finding	Reference
		Dose (mg/kg/day)	Route	Duration		
Kunming mice	8	1250	IP	8 weeks	SOD ↓	Wang et al. [61]
C57BL/6 mice	8	800	IP	60 days	MDA ↑, SOD ↓, GSH ↓, CAT ↓	Yang et al. [62]
Kunming mice	8	500	SC	11 weeks	MDA ↑, SOD ↓, CAT ↓, GSH-Px ↓	Zhao et al. [63]
ICR mice	9–11	500	SC	6 weeks	MDA ↑, SOD ↓, CAT ↓	Liu et al. [64]
Kunming mice	/	400	SC	58 days	MDA ↑, SOD ↓, GSH-Px ↓	Wang et al. [65]
Kunming mice	8	200	SC	8 weeks	MDA ↑, SOD ↓, CAT ↓, GSH-Px ↓	Mo et al. [66]
C57BL/6 mice	5	150	SC	10 weeks		Nam et al. [67]
ICR mice	10–12	120	IP	12 weeks	MDA ↑, SOD ↓, CAT ↓, GSH-Px ↓	Liu et al. [68]

Note: IP, intraperitoneal; SC, subcutaneous; MDA, malondialdehyde; SOD, superoxide dismutase; CAT, catalase; GSH-Px, glutathione peroxidase; GSH, glutathione; CAT, catalase

Table S2. Effects of D-gal on concentrations of CAT and MDA in the liver of male mice.

Group No.	Dose (mg/kg/day)	CAT (U/mgprot)	MDA (nmol/mgprot)
1	0	15.36 ± 0.99^{a}	29.85 ± 2.66^{a}
2	120	13.37 ± 2.32^{a}	31.89 ± 1.01^{a}
3	400	6.48 ± 1.01^{b}	35.09 ± 0.11^{b}
4	800	2.71 ± 0.04^{c}	39.17 ± 1.18^{c}

Data are presented as the mean ± SD (n = 8). Mean values with different superscripts (a, b, c) in the same column are significantly different ($P < 0.05$), whereas the same superscripts are not ($P > 0.05$).
